# NO_X_ious gases and the unpredictability of emerging plant pathogens under climate change

**DOI:** 10.1186/s12915-017-0376-4

**Published:** 2017-05-08

**Authors:** Helen N. Fones, Sarah J. Gurr

**Affiliations:** 1 Biosciences, University of Exeter, Stocker Road, Exeter, EX4 4QD UK; 2 Rothamsted Research, North Wyke, Okehampton, EX20 2SB UK; 3 Donder’s Hon Chair, University of Utrecht, Padualaan 8, 3584 CH Utrecht, The Netherlands

## Abstract

Emerging pathogens of crops threaten food security and are increasingly problematic due to intensive agriculture and high volumes of trade and transport in plants and plant products. The ability to predict pathogen risk to agricultural regions would therefore be valuable. However, predictions are complicated by multi-faceted relationships between crops, their pathogens, and climate change. Climate change is related to industrialization, which has brought not only a rise in greenhouse gas emissions but also an increase in other atmospheric pollutants. Here, we consider the implications of rising levels of reactive nitrogen gases and their manifold interactions with crops and crop diseases.

## Emerging pathogens

An emerging plant pathogen (EPP) is the causative agent of a new disease, an infection on a novel host, or a pathogen in an extended geographic range. EPPs arise as a result of the continuous evolutionary arms race between host and pathogen. We have, however, recently witnessed an intensification in the rate at which EPPs arise, bringing with it increased threats to natural and agricultural ecosystems [1–4]. Much of this can be attributed to anthropogenic changes: agro-ecosystems composed of monocultures, or which provide year-round host availability, are a unique cradle for the evolution of crop diseases [5, 6]. Meanwhile trade helps to spread fungal inoculum worldwide [4].

A well-known example of the impact of an EPP is seen in the loss of millions of elm trees due to *Ophiostoma novo-ulmi* (Dutch Elm disease) in the 1970s [7]. Currently, *Hymenoscyphus fraxineus*, spread by trade in infected, asymptomatic seedlings, is causing serious ash dieback outbreaks in Europe [8, 9]. The keystone, pioneer Hawaii’n species ‘Ōhi’a (*Metrosideros polymorpha*) is threatened by *Ceratocystis fimbriata*, a fungal pathogen causing a new disease: rapid ‘Ōhi’a death [10]. There are also well-publicized examples of emerging fungal disease in animals such as amphibians, bats, and bees [1].

**Fig. 1.**
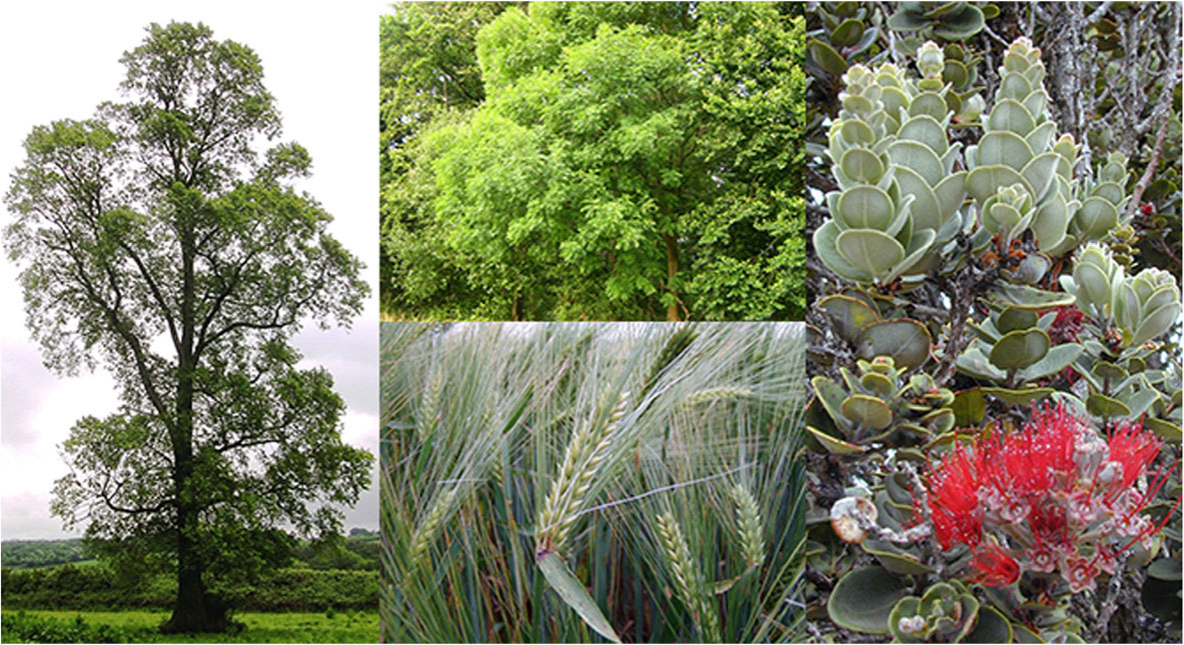
Examples of species affected by EPPs. Clockwise from left: English Elm (*Ulmus minor*) (photograph by Ptelea [136]), decimated by the “Dutch Elm disease” pathogen, *Ophiostoma novo-ulmi*; European Ash (*Fraxinus excelsior*; photograph by Botaurus stellaris [137]), under threat from “Ash dieback” caused by *Hymenoscyphus fraxineus*; ‘Ōhi’a (*Metrosideros polymorpha*; photograph by Forest & Kim Starr [138]), threatened by “Rapid ‘Ōhi’a death” due to *Cerasystis frimbriata* and barley (*Hordeum vulgare*; photograph by raul.dupagne [139]), the host of *Ramularia collo-cygni*

Given that agro-ecosystems provide excellent conditions for the emergence of new diseases and that crop disease is of economic concern (for example, [11]), it is unsurprising that many well-studied examples of emerging pathogens come from agriculture. These include *Ramularia collo-cygni*, a European barley pathogen which emerged in South America in 2011 and which has also recently developed virulence against oats and wheat [12, 13] (Fig. 1). *Magnaporthe oryzae,* primarily known as rice blast disease, was detected on wheat in Brazil in 1985 and Bangladesh in 2015–16 [14, 15]. The generalist charcoal rot root pathogen *Macrophomina phaseolina* recently emerged as a disease of soybean in both the USA and Africa’s Sahel [16]. *M. phaseolina* is most problematic in hot, arid conditions and is expected to spread to new regions under most climate change scenarios [17].

## Impact of climate change on plant pathogens

Changes in global climate are generally projected to comprise an increase in global average temperatures of around 1–2 °C by 2100, alongside an increase in the frequency of extreme events including periods of high temperature, storms, or drought [18–20]. These changes will influence pathogen emergence in new areas; these influences are discussed in the following sections. Climate alters the susceptibility of the host, and influences the host cultivars planted. It also drives the distribution of hosts, both cultivated and wild, alters trade patterns, and determines ranges of competitor and biocontrol species [21, 22]. Climate also affects the virulence of many pathogens [17]. In response to these changes in climate and their direct and indirect effects on crops and their pathogens, new crop varieties may be developed. This process currently takes around 20 years on average [23], however, so that while adaptation is possible it may be outpaced by continuing, rapid change.

## Interactions between climate and pathogen virulence

The impact of combined abiotic and biotic stresses on plants, imposed by a changing climate, will also alter host susceptibility to EPPs (for example, [24–26]). Such interactions take place at various levels, including early signaling hubs such as calcium-dependent and mitogen-activated protein kinases (for example, [27, 28]), reactive oxygen (ROS) signaling [29], plant hormones (for example, [30]), and other signaling molecules (for example, [31]). These signals modulate gene transcription, cell biology, and physiology to respond to multiple stresses appropriately [24]. Both cross-protection and cross-vulnerability between stresses have been documented in plants: salt stress enhances barley resistance to *Blumeria graminis* [32] and drought protects tomatoes against *Botrytis cinerea* [33], but drought-resistant, aerobic rice varieties are more susceptible to root-knot nematodes [34] and drought stress renders beans more vulnerable to *M. phaseolina* [35]. Plant defense itself is not static, but exists in the context of rapidly evolving pathogen populations [36, 37], which will also respond to climate variations [38, 39].

Among pathogens, Sturrock et al*.* [40] describe two groups. The first group infects stressed plants and will become more problematic under climate change-induced stress. Examples include *M. phaseolina* and canker diseases of trees caused by opportunistic fungi, including the emerging pathogen of oak, *Biscogniauxia mediterranea*. This fungus was first described in Mediterranean oaks; however, it emerged in Slovenia in 2006 [41] following low rainfall and above average temperatures. As increased aridity spreads north, this pathogen is expected to follow [42].

## Interactions between climate and pathogen distribution

The second group of pathogens infects healthy plants, under favorable environmental conditions. These pathogens are directly influenced by climate and weather, for instance requiring warm, wet conditions to infect. An emerging pathogen in this group is *Phytophthora ramorum* (sudden oak death). This oomycete relies upon moist, mild winters for survival and on rainfall for transmission [43]. Since 2009, it has become problematic in Southwest England on larch, as well as emerging on a number of new hosts [44–46]. This correlates with increased incidence of winter rainstorms in this area [47].

In common with *P. ramorum*, many pathogens are expected to change their ranges as a result of climate change (for example, [48, 49]). Recently, Bebber et al*.* [50] used historical disease data to show that crop pests and pathogens have been moving towards the poles at an average of 2.5–3 km per year since 1960, as global temperatures have increased. This rate is as high as 6–7 km per year for fungi and oomycetes. A number of studies have attempted to model future pathogen distributions using either climate matching [51, 52] or process-based approaches [53, 54]. Both have limitations, and it can be hard to test their underlying assumptions [55]. Some authors argue that predictions of pathogen spread and impact are too uncertain to have a role in shaping policy, and that we are better served by preparing for multiple scenarios [55–57]. This view is based upon the paucity of present and historical disease occurrence data, difficulties with modeling and extrapolating into the future, and complexities inherent in understanding how a prediction of pathogen emergence translates into risk of yield loss.

The greatest factor driving pathogen distributions is the distribution of the host [4, 5], followed by the distribution of alternative hosts and the availability of susceptible hosts throughout the year [4, 5, 58]. There is evidence that saturation of hosts by pathogens should be expected in the long term [59], and that the speed at which this happens is largely dependent on trade and transport of hosts and host products [59, 60]. The accuracy of disease predictions thus depends upon the ability to predict trade, land use and crop choice, all of which may depend, themselves, upon climate and disease incidence.

Individual, case-by-case studies are perhaps more likely to yield useful predictions, having more specific parameters. For example, models predict that occurrence and severity of *Fusarium* head blight of wheat (FHB) are likely to reach a 30 year high in South American wheat growing regions as a result of increased rainfall under climate change [61], while FHB epidemics severe enough for mycotoxins to exceed safe levels are expected in the UK by 2050 [55, 62].

Another disease expected to show an expanded range under climate change is oak decline. The causal agent, *Phytophthora cinnamomi*, a soil-borne pathogen that requires warmth and moisture, infects *Quercus* species in southern Europe, extending north along the west coast of France*.* Initial studies using the CLIMEX model for species distribution predicted that oak decline would become more severe, due to elevated warmth and moisture promoting pathogen growth and transmission, but would not spread north or east, due to lower winter temperatures [63]. Later work, however, which considers the micro-climate within oak phloem, where this pathogen is found, suggests that the disease may spread hundreds of kilometers eastward [64]. This contradiction highlights the difficulties in predicting the behavior of even a single pathogen in a specific location.

Climate change is not simply gradual increases or decreases of temperature or rainfall, but results in unpredictable short-term changes in weather and extreme weather events. These can alter the likelihood of the spread, establishment and epidemics of pathogens in new areas. Citrus canker, a water-droplet borne bacterial disease, became irrevocably established in Florida after four hurricanes made landfall during 2004 [65], and outbreaks of *Dothistroma* needle blight have been found to correlate with above average rainfall events in British Colombia [66]. Morley and Lewis [67] studied the effects upon pathogens of the drought which affected the UK in 1976. In fungi, these effects were dependent upon pathogenic lifestyle: many foliar pathogens were less successful in producing aerial spores, while soil-borne species were largely unaffected [67]. Anyamba et al*.* [68] attempted to characterize the contribution of extreme weather events to disease outbreaks globally, and attributed 10–80% variation in agricultural productivity to weather extremes [68, 69]. Taken together, these examples illustrate the importance of the inherently unpredictable result of climate change upon weather.

## Changing atmospheric composition

Anthropogenic climate change occurs due to production of greenhouse gases (GHG), especially CO_2_ [19, 70, 71]. The increase in CO_2_ production can be largely attributed to increasing industrialization [70, 71]. Fundamentally, increases in GHG production are due to increased energy use, but can also be attributed to changes in land use, including deforestation, urbanization, and agricultural intensification [72, 73]. The rising global demand for meat [74, 75] has also led to higher CO_2_ emissions [76]. Greater use of agricultural inputs of fertilizer have also been implicated in increased GHG emissions [77].

These changes are responsible for the production of a range of atmospheric pollutants, some of which contribute to climate change alongside CO_2_ and all of which affect life on the planet. Since pre-industrial times, there has been a sharp and accelerating increase in levels of the major, long-lived GHGs CO_2_, CH_4_ (methane), and N_2_O (nitrous oxide) [18, 78, 79]. Gases such as NO and NO_2_, produced during fossil fuel combustion, also affect GHG accumulation via atmospheric reactions which produce GHGs or alter their half-lives [80].

## Case study: nitrogenous gas pollutants

Due to the rising human population, both energy use and global food requirements have risen. Whilst crop production has increased steeply since the Green Revolution [81], it must continue to increase if we are to meet future needs. A portion of the increase in yields has been due to a rise in agricultural inputs. Most notably, from 1950–2000 the use of nitrogen fertilizers increased more than 20-fold [82]. This was made possible by the development of the Haber–Bosch process for fixing atmospheric nitrogen to ammonia. Industrial production of nitrogen fertilizers began in the early 20th century [82, 83] and has been fundamental to food security ever since, as nitrogen availability is often the limiting factor in crop productivity [84]. Up to 90% of the nitrogen inputs into agriculture are, however, lost—as NH_3_, NO_2_, or NO [82, 84]. These gases have a number of effects upon crop productivity, climate change, and pathogen emergence.

**Fig. 2.**
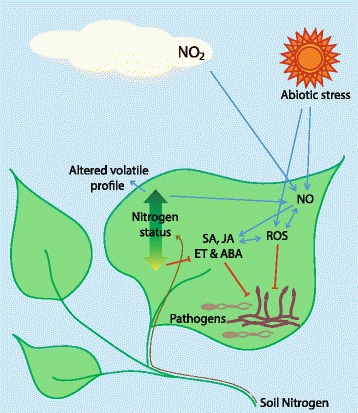
Schematic representation of some of the interactions between plant nitrogen status and defense signaling. In concert with reactive oxygen species (ROS), NO acts to mediate plant hormone signaling and anti-pathogen defenses. Atmospheric NO_2_ can enter leaves and stimulate the production of NO, while abiotic stress interacts with biotic stress signaling via both ROS and NO. In addition, plant nitrogen status can impose restraints on defense signaling when low and promote NO production if high. Factors including root features, soils, and soil ecology that may affect plant nitrogen status are not represented in this figure

Since production of these nitrogenous gases is correlated with human population, energy use, and, therefore, with the emissions—such as CO_2_ and methane—that underlie climate change, prediction of crop disease under climate change requires that their many possible interactions with pathogens and hosts alike are effectively incorporated into models describing climate change scenarios. Anthropogenic changes in the composition of the atmosphere that may influence plant–pathogen interactions are summarized in Fig. 2. A first step to building such models would be to understand what happens to the nitrogen lost from agro-ecosystems. Nitrates are often lost as runoff, but other nitrogenous waste products are lost to the atmosphere where, eventually, they revert to N_2_ [84]. Anthropogenic nitrogen inputs are not limited to fertilizer application, but include increased deposition of nitrogen as a result of its increased atmospheric concentrations and resulting changes to the nitrogen cycle [83]. The rate of reactive nitrogen (NO_x_, NH_4_, and NH_3_) emissions currently exceeds the rate of N_2_ formation, meaning that nitrogen is building up in the atmosphere and in ecosystems [84]. NO_x_ and NH_3_ emission and deposition rates are currently over ten times greater than those occurring naturally, and are expected to exceed levels thought to be ecologically safe by 2050 [83]. Reactive nitrogen can be metabolized by many soil microbes, and can thus alter the soil and rhizosphere microbiomes, which will in turn affect soil microfauna and plants [84, 85]. Like the effects of CO_2_ emissions, nitrogen deposition does not necessarily take place in the same geographical area as emission; rates of deposition are greatest in vulnerable tropical and forest ecosystems and agro-ecosystems [83, 86, 87].

Increased reactive nitrogen in the atmosphere is not merely correlated with the causes of anthropogenic climate change, but also has its own effects upon climate. NO_x_ emissions can lead to formation of ozone, a GHG that can reduce CO_2_ uptake by plants [88, 89]. Increased nitrogen deposition may promote methane production by soil microbes [90], but may also promote plant growth, thus acting as a carbon sink. This is barely scratching the surface of the complexity of interactions between atmospheric nitrogen, plants, and climate. Pathogens, their responses to nitrogen, both direct and indirect, and the downstream effects upon the hosts, ecosystems, emissions of carbon and nitrogen, and the effect of climate change resulting from those emissions on the pathogens and their hosts provide a circle—or perhaps spiral—of complex interacting factors and outcomes.

## Effect of nitrogen on host plants

Nitrogen can alter the susceptibility of plants to disease in two major ways. Firstly, plants’ nitrogen status may impact on their nutritional value to pathogens; secondly, nitrogen affects plant biotic and abiotic stress responses. Altered abiotic stress responses naturally alter plant responses to changes in climate, but also influence responses to pathogens, through crosstalk with biotic stress signaling.

## Host nitrogen nutrition

Nitrogen is an essential element for both plants and microbes. Many pathogens express pathogenicity factors when nitrogen is limiting [91, 92], suggesting that this situation is common during infection. Thus, it might be expected that increased nitrogen availability in the ecosystem, due to increasing atmospheric nitrogen concentrations and resultant increases in nitrogen deposition rates, would lead to increased host nitrogen status and would promote disease development [93]. Indeed, *Uncinula necator*, the cause of grapevine powdery mildew, is more successful when host plants have high nitrogen status, a finding partially attributed by Keller et al*.* [94] to a resultant increase in nitrogen and sugars in host tissues. By contrast, however, the fungal pathogens *M. oryzae* and *Fusarium oxysporum* are less virulent when supplied with their preferred nitrogen source [95]. In fact, the relationship between plant nitrogen status and disease incidence is nuanced. Both positive and negative correlations have been reported [95, 96] and there is evidence that the response of the pathogen might depend upon its lifestyle, with biotrophic organisms favored when the host is nitrogen replete and necrotrophs when the host is nitrogen limited [95, 97].

One complication is that the presence of pathogens affects the plants’ nitrogen metabolism through both altered nitrogen sinks in infected tissues and plant defense activities evolved to deprive the pathogen of nutrients [98]. Another is that altered nitrogen status and resulting metabolic changes can affect many aspects of plant physiology, with direct or indirect consequences for pathogens: *Brassica napus* produces altered floral volatiles after nitrogen fertilization, including an increase in acetic acid, an antifungal to which a correlated fall in *Alternaria* dark spot disease was attributed [99]. Similarly, the grapevines studied by Keller et al*.* [94], which were more susceptible to powdery mildew when grown on increased nitrogen, also showed reduced levels of defensive polyphenols under these conditions. Further, low nitrogen availability may lead to reduced investment in defenses, with lower activity of the anti-fungal enzymes chitinase, chitosanase, and peroxidase in *Arabidopsis* under such conditions [100]. The *Arabidopsis* nitrogen receptor and transporter NRT2.1 also represses salicylic acid (SA)-dependent anti-biotroph defenses in response to low nitrogen, increasing susceptibility to *Pseudomonas syringae* [101]*.*

Nitrogen uptake, amino acid metabolism [98], and responses to nitrogen status may also be altered in infected plants [92, 96, 102–104]. Glutamine synthase gene expression, for example, is upregulated in tobacco and *Arabidopsis* [95] in response to pathogens. As well as changes to host nitrogen metabolism following pathogen detection, there are changes induced by the pathogen. Pathogens seek to hijack plant metabolism, using effectors, toxins, and strategic metabolite use and production to achieve this [95–97, 102, 105]. There is evidence that regulation of amino acid metabolism is therefore important in plant defense [95, 96].

## Plant stress signaling

Increased atmospheric NO_x_ does not only affect plants after being deposited into their ecosystem; it also has more direct effects upon plant growth. Generally, NO and NO_2_ pollution leads to reduced growth of plants. This is because, as well as interacting with other atmospheric molecules with potentially deleterious results, both act as toxins [106]. NO_2_ is taken up by foliar tissues via the stomata [107], and, at low concentrations, can act as a nitrogen fertilizer, being largely translocated to the root [108, 109] and decreasing carbon/nitrogen ratios [110]. Although, as discussed above, these changes in nitrogen nutrition may have knock-on effects for plant–pathogen interaction, the real significance of foliar NO_2_ absorption comes from its induction of reactive nitrogen within the leaf.

On pathogen challenge, plants rapidly produce a high concentration of reactive oxygen species (ROS), known as the “oxidative burst”, which acts both as a direct antimicrobial and in defense signaling [111]. In the last 30 years, it has been realized that reactive nitrogen species (RNS) have a similar role, with a nitrosative burst accompanying the oxidative [112–114]. RNS produced during this process include the nitrogen oxide and dioxide radicals (NO and NO_2_), the nitric oxide anion (NO^−^), the nitrosonium ion (NO^+^), peroxynitrite (OONO^−^), and nitrothiols (SNOs) [115, 116].

Importantly, RNS and ROS signaling occur in plants in response to exogenous NO_2_ gas [117]. High levels of exogenous NO_2_ are phytotoxic and can induce stunting, cell death, lipid peroxidation, production and activation of ascorbate, glutathione, and anti-oxidant enzymes, protein modifications, and the induction of SA, jasmonic acid (JA), and abscisic acid (ABA) signaling [106, 117, 118]. All of these downstream effects of NO_2_ exposure are either directly involved with, or have the capacity to interact with, defense signaling, although the roles of factors such as plant hormones and antioxidants in plant stress signaling have been reviewed elsewhere and are too large a topic to summarize here.

Uptake of atmospheric NO_2_ by foliar tissues shows a linear relationship to its concentration [119]. Once inside the leaf, NO_2_ is rapidly solubilized into the apoplast, forming NO_2_^−^, NO_3_^−^, and H^+^ ions [119]. It has been assumed that the reason that NO_2_ is phytotoxic is that nitrite ions are transported to the chloroplast, where they reduce stromal pH and compete for NADP^+^, reducing carbon fixation [106]. Indeed, photosynthesis is sensitive to NO_2_ [120]. Reduced photosynthetic capacity following this toxicity is likely to lead to energy conserving down-regulation of defenses, as seen for low nitrogen status. More important in the current discussion, however, is the role of NO_2_ in increasing the foliar concentrations of NO. This can occur in at least two ways. Firstly, as is well known in inorganic systems, NO_2_^−^ spontaneously evolves NO at low pH, a process greatly enhanced by the presence of a reductant such as ascorbate [120]. This may occur, for instance, in the mildly acidic apoplast where NO_2_ first dissolves to form NO_2_^−^, and where ascorbate is an important antioxidant following the uptake of this gas [121]. Secondly, nitrate reductase in the chloroplasts can produce NO from NO_2_, using NADPH as an electron donor, accounting for the competition effect with respect to NADPH [120]. This pathway for NO production from NO_2_ absorbed via the stomata has been demonstrated to occur commonly [117, 120].

Once produced, NO and its ions are well-adapted as signaling molecules, diffusing easily throughout the cytoplasm and across lipid bilayers [122]. NO is implicated in many aspects of plant physiology and development, including pollen tube growth, germination, leaf and root organogenesis, cell wall lignification, flowering, fruit ripening, and senescence [115, 122]. It is a signal of abiotic stresses, including drought, salt, high light intensity and UV, high and low temperatures, wounding, ozone, and heavy metals [115, 122, 123]. NO also signals biotic stresses such as pathogen attack [115, 122] or establishment of plant-microbe symbioses [124, 125], a process that initially elicits many elements of the plant defense response, later suppressed by compatible symbionts [126]. As with ROS signaling, it is emerging that pathogens also rely on RNS in development and as a virulence factor [127, 128] and that plant RNS signaling can be hijacked by pathogens [128], as well as by beneficial micro-organisms [129]. Taken together, the various signaling roles of RNS in plant–microbe interactions develop as complex a picture as do the roles of ROS (for example, [130, 131]).

During the oxidative and nitrosative bursts, RNS can react with ROS. For example, peroxynitrite is formed when superoxide meets nitrogen oxide. Thus, the two sets of reactive molecules can modulate each other’s concentrations and signaling functions. NO increases the antioxidant capacity of plant cells, protecting the plant from its own ROS response to stressors, including pathogens, UV, and salt [115, 122, 132]. NO also interacts with various plant hormone pathways, including auxins, cytokinins, SA, JA, and ethylene (ET) [133, 134]. NO can interact with DNA and transcription factors directly, and S-nitrosylation of transcription factors and other proteins can lead to altered gene expression and protein activities [115, 134, 135]. As a result of these abilities, NO can influence the SA signaling pathway at various stages from upregulation of SA biosynthesis, to nitrosylation of transcription factors that facilitate the expression of SA-dependent genes, to nitrosylation of NPR1, promoting oligomerization of this protein and its retention in the cytoplasm, which, by contrast, reduces SA-dependent gene expression [133]. Reactive nitrogen thus is recognized as a key factor in SA signaling, systemic acquired resistance, and the hypersensitive response [115, 134]. In addition, interaction of NO with other defense-related phytohormones can add further layers of modulation to the plant defense response [133].

**Fig. 3.**
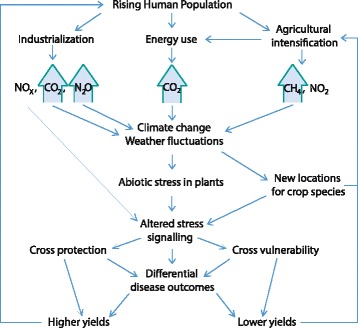
Schematic representation of the various factors that may impact plant–pathogen interactions via anthropogenic changes in atmospheric composition

Induction of NO accumulation and the complex signaling activities of reactive nitrogen as a result of exposure to atmospheric NO_x_ means that all of the above interactions are relevant to our understanding of how both plants and their pathogens will react to changing atmospheric composition. As detailed here, these interactions are multi-layered and extraordinarily complex (Fig. 2). This means that precise predictions of impact and risk will be difficult to generate, but nevertheless, some general points can be made. As the human population increases, leading to greater energy expenditure and to reliance upon ever more intensive agricultural systems, we can expect an amplification of risk from certain crop pathogens. As atmospheric CO_2_ concentrations increase, we expect temperatures to increase in combination with higher or lower humidities in different areas, along with increased risks from unpredictable events like flooding and droughts. These abiotic stresses will combine with atmospheric NO_x_ and other factors to alter plant stress signaling and thus susceptibility to disease (Fig. 3). Outcomes are likely to depend heavily upon pathogen lifestyle, which may alter the prevailing risks in a climate- and nitrogen deposition-dependent manner. Due to agricultural intensification and the fact that intensive agro-ecosystems and agricultural trade favor the evolution and dissemination of pathogens, it is likely that any group of pathogens favored in a given area will emerge as a serious threat to the food-security of that region. Mitigation of disease risk is likely to depend upon broad and flexible contingency planning; the most useful models are likely to be those built on a case-by-case basis, incorporating well-studied hosts and pathogens in specific geographical regions.
